# Non-recurrent right laryngeal nerve identified during endoscopic thyroidectomy via areolar approach: a case report

**DOI:** 10.3389/fsurg.2023.1272431

**Published:** 2023-10-06

**Authors:** Xiaohu Jin, Ronghua Yuan

**Affiliations:** Department of Thyroid and Breast Surgery, Nantong City No 1 People’s Hospital and Second Affiliated Hospital of Nantong University, Nantong, China

**Keywords:** nonrecurrent laryngeal nerve, recurrent laryngeal nerve, endoscopy, thyroidectomy, case report

## Abstract

**Background:**

Nonrecurrent laryngeal nerve (NRLN) is a rare but significant anatomical variation in thyroid surgery, and lack of awareness of NRLN may lead to intraoperative injury. Here, we report a clinical case of NRLN discovered during endoscopic thyroid surgery via total areola approach in a 23-year-old female patient.

**Case presentation:**

A 23-year-old female patient presented with bilateral thyroid nodules for three years. She underwent bilateral thyroid nodule fine-needle aspiration biopsy and BRAF gene testing at our hospital, with results indicating bilateral papillary thyroid carcinoma and positive BRAF gene V600E mutation. Neck-enhanced CT revealed bilateral thyroid nodules and the right subclavian artery branching from the aortic arch on the distal side of the left subclavian artery. The patient underwent endoscopic thyroidectomy via total areola approach for radical resection of bilateral thyroid cancer. Intraoperatively, NRLN was found on the right side and RLN on the left side. The surgery was successful, and no postoperative complications were observed. Postoperative pathology confirmed bilateral papillary thyroid carcinoma.

**Conclusions:**

Although NRLN is a rare occurrence, clinicians should not overlook its presence to prevent serious complications. Preoperative imaging confirmation of the presence or absence of an abnormal subclavian artery course is crucial in preventing the sudden discovery of NRLN during surgery. Endoscopic thyroid surgery via total areola approach is a safe and effective technique but requires a high level of professional skills and an understanding of anatomical variations to prevent nerve injury.

## Introduction

NRLN is a rare variation of the recurrent laryngeal nerve, with the estimated incidence rate of NRLN on the right side being relatively low (0.2%–1.5%), and even lower on the left side (0.04%). The overall incidence rate is only 0.7% (1–3). The formation of NRLN is mainly related to abnormal arterial development during embryonic development, such as the maldevelopment of the aortic arch or the brachiocephalic trunk. This may explain why NRLN is more common on the right side and often associated with subclavian or lusoria artery anomalies. During thyroid surgery, the laryngeal recurrent nerve is of considerable importance to the surgeon, so much so that surgical strategies are continuously being optimized to avoid complications such as hoarseness or dysphagia (4). NRLN originates directly from the vagus nerve and does not recur into the mediastinum, which increases the difficulty and risk of complications during surgery. Therefore, accurately identifying the course of NRLN and protecting it is crucial during surgery.

## Case presentation

**Figure 1:**
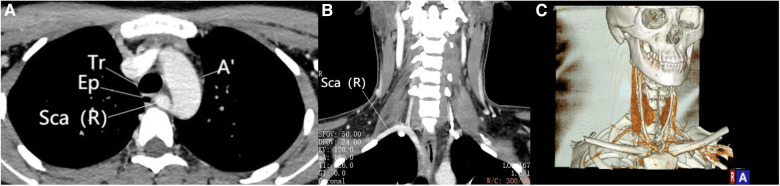
Enhanced CT scan. (**A**) Viewed in axial view, the right subclavian artery originates from the aortic arch and courses between the vertebrae and the esophagus. (**B**) Viewed in coronal view, the ectopic course of the right subclavian artery can be observed. (**C**) The blood vessels under three-dimensional reconstruction can be observed. Sca (R), Right subclavian artery; Ep, Esophagus; Tr, Trachea; A, Aorta.

**Figure 2:**
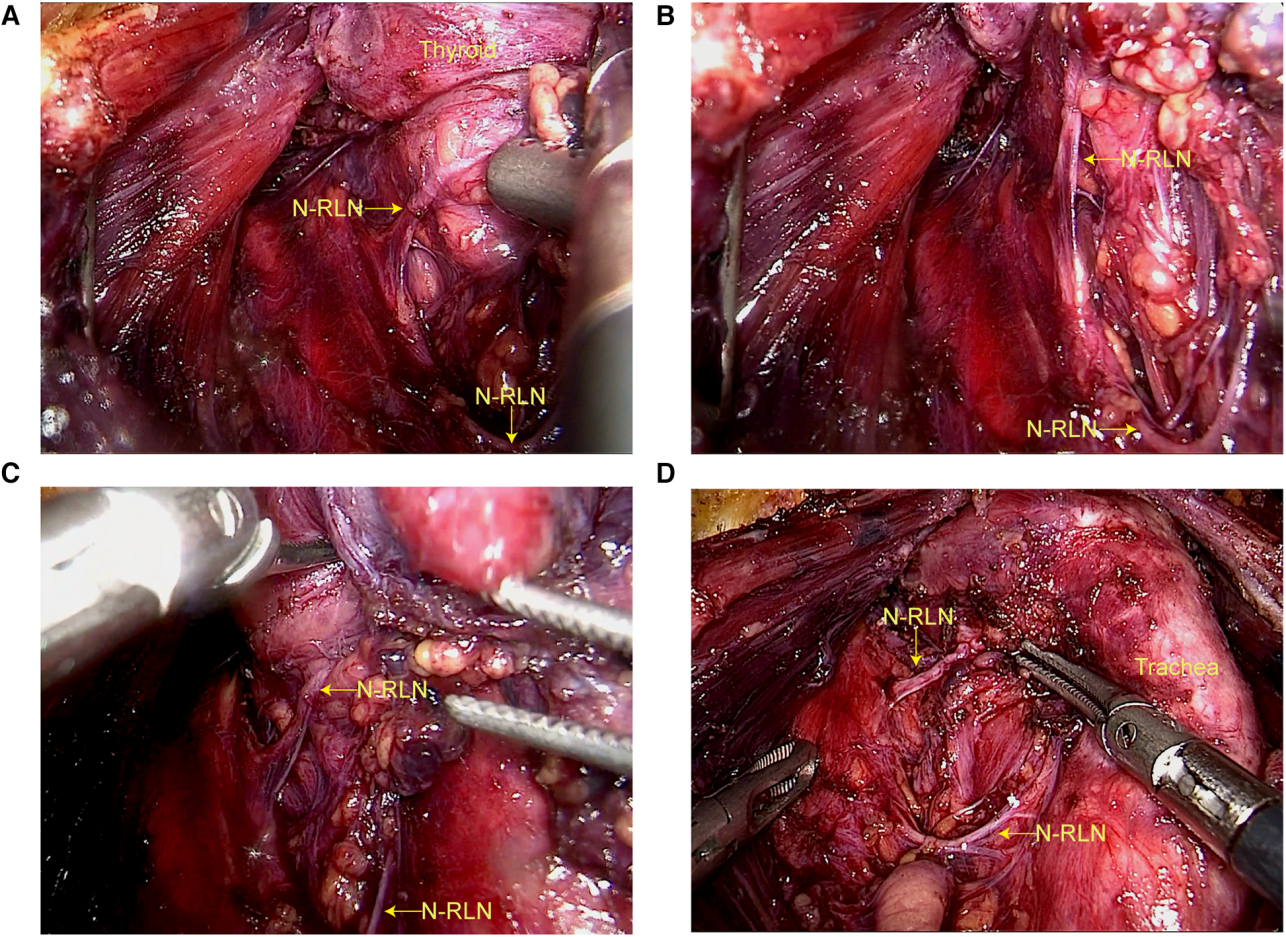
Right nonrecurrent laryngeal nerve during endoscopic thyroidectomy via areolar approach. (**A**) Mistakenly identified as the superior thyroid artery, the NRLN. (**B**) Due to preoperative confirmation of anomalous course of the subclavian artery, the NRLN, which was mistakenly identified as the superior thyroid artery, was carefully dissected along its course instead of being transected. (**C**) Found to course through the thyroid gland and into the larynx. (**D**) Ultimately confirmed to be the NRLN.

In March 2023, a 23-year-old female patient presented to our hospital's thyroid surgery department with a history of bilateral thyroid nodules detected during a routine physical examination for 3 years. She had no known medical history or chronic diseases and no hoarseness or compression symptoms. Thyroid function, anti-peroxidase antibody, and calcitonin levels were all within normal limits. Ultrasound showed a solitary solid nodule measuring 2.5 × 2.0 mm with unclear margins and a regular shape in the left thyroid lobe, with a longitudinal-to-transverse ratio greater than 1. A solitary solid nodule measuring 5.5 × 3.5 mm with unclear margins and a regular shape, with punctate calcification inside, was found in the right thyroid lobe. No abnormal lymph nodes were found in the neck. The patient underwent bilateral thyroid nodule fine-needle aspiration biopsy and BRAF gene testing, with results indicating bilateral papillary thyroid carcinoma and positive BRAF gene V600E mutation. Neck-enhanced CT revealed bilateral thyroid nodules and the right subclavian artery branching from the aortic arch on the distal side of the left subclavian artery (Figure 1). Due to the patient's young age and aversion to a midline neck incision, she underwent endoscopic thyroid surgery via total areola approach for radical resection of bilateral thyroid cancer, including total thyroidectomy and ipsilateral and contralateral central compartment VI lymph node dissection. During the surgery, a small nerve was discovered that wrapped around the inferior thyroid artery and passed upward along the tracheoesophageal groove on the right side. Further dissection revealed a cord-like grayish-white structure emanating from the neck sheath and extending horizontally to the posterior aspect of the thyroid gland. Careful dissection along this cord-like structure revealed a nerve running horizontally inward to the thyroid cartilage below the inferior horn, which was identified as NRLN (Figure 2). The postoperative period was uneventful, with no changes in the patient's voice and no numbness in the extremities. According to the postoperative pathological examination, the patient was found to have bilateral micro-papillary thyroid carcinoma.

## Discussion

NRLN is a rare developmental anomaly that was first discovered during a cadaver dissection by Stedman in 1823 (5). NRLN on the right side is usually associated with an abnormal subclavian artery, which is often referred to as the right vagus below the clavicle artery or “arteria lusoria”, and is usually due to the regression of the fourth pharyngeal arch during embryonic development. NRLN on the left side is often associated with complete visceral situs inversus (6). NRLN can be classified into different types based on its course, with the Toniato classification (7) system being commonly used. Type I NRLN originates directly from the vagus nerve trunk in the neck, accompanying the superior thyroid artery and descending into the larynx. Type IIA NRLN arises from the recurrent laryngeal nerve trunk at the level of the inferior thyroid artery and runs parallel to the inferior thyroid artery into the larynx. Type IIB NRLN arises from the recurrent laryngeal nerve trunk at the level of the inferior thyroid artery and runs parallel to the inferior thyroid artery into the larynx, passing between the inferior thyroid artery and its branches before entering the larynx. Our case does not fit into either Type I or Type IIA, but rather appears to be a combination of Type I and Type IIA NRLN. To date, only Mohammed AA (8) has reported a case in which 2 ipsilateral NRLN (double nerves) were found to cross over and parallel to the inferior thyroid artery at the time of a routine right thyroid lobectomy. The presence of NRLN increases the risk of nerve injury during thyroidectomy. However, NRLN is not visible on any imaging examinations. Therefore, NRLN is often “accidentally” discovered during surgery and may sometimes be overlooked or inadvertently injured. Accurate prediction of NRLN before surgery is crucial for avoiding nerve injury during surgery.

Esophageal barium meal examination may reveal the compression of the esophagus by the abnormal right subclavian artery, which can create a circular notch on the left edge of the esophagus. However, this is not a routine preoperative examination for patients with thyroid disease, so it has certain limitations in diagnosing NRLN. Ultrasound is the preferred preoperative auxiliary examination for patients with thyroid disease and can also be used to diagnose NRLN by evaluating the presence of vascular malformations. Normally, the right common carotid artery and right subclavian artery originate from the brachiocephalic trunk. Under ultrasound, this structure is called the “Y sign”. When both the right common carotid artery and right subclavian artery originate separately from the aortic arch, the “Y” sign disappears under ultrasound, indicating the absence of the brachiocephalic trunk and the possible presence of NRLN (9). However, sometimes ultrasound cannot detect the branching of the brachiocephalic artery due to the pseudo-image of thyroid nodules and the clavicle, which is a limitation of ultrasound in identifying NRLN. Preoperative CT has a high detection rate for NRLN (10). CT can not only understand the anatomical relationship between the thyroid and surrounding tissues and the presence of lymph node metastasis in the neck but also discover the absence of the brachiocephalic trunk. Three-dimensional reconstruction technology can also improve the detection rate of NRLN. If the three-dimensional reconstruction shows that the right subclavian artery originates from the distal aortic arch of the left subclavian artery and crosses the midline behind the esophagus to the anterior cervical vertebrae on the right side, then the presence of right NRLN should be considered. Therefore, CT and ultrasound images can be used for preoperative diagnosis of NRLN. As in our case, neck-enhanced CT identified the abnormal position of the right subclavian artery, suggesting the presence of right NRLN. Knowing the presence of anatomical variation before surgery and the possibility that the RLN nerve may not be located in the tracheoesophageal groove before surgery, the discovery of NRLN during surgery is not surprising.

Intraoperative neural monitoring (IONM) has been widely used for identifying and monitoring the recurrent laryngeal nerve (RLN) during thyroid and parathyroid surgery. The use of IONM is beneficial for preventing RLN and NRLN injury. Demet Sengul et al. (11) reported a case of sutureless total thyroidectomy with intermittent intraoperative neuromonitoring (I-IONM) and energy-based instrumentation without sternotomy for the treatment of retrosternal goiter with chronic lymphocytic thyroiditis, demonstrating that IONM provides a strong barrier for complex thyroid surgery. Using IONM during surgery resulted in a lower incidence of nerve paralysis, especially in cases involving NRLN (12). In this case, it was fortunate that the NRLN could be identified despite the absence of an IONM, which was also attributed to the preoperative CT identification of the aberrant right subclavian artery as well as the magnified field of endoscopic thyroidectomy. Whether thyroidectomy (13), endoscopic thyroidectomy (14) or robotic thyroidectomy (15), the IONM can be used to guide the precise dissection of the NRLN as well as improve safety. As technology has evolved, new methods of neuromonitoring have also emerged. Iyad Hassan et al. (16) used and confirmed a new neural monitoring method, Abu Dhabi Neural Mapping (ADNM), for early identification of NRLN issues and preservation of its function during minimally invasive thyroidectomy.

Despite the help of IONM, (abnormal) anatomical knowledge should also be a core component of medical students and surgical interns' skills (17). A comprehensive understanding of the normal and abnormal anatomical structures of RLN is still crucial for the optimal results of thyroid surgery. Boyan Fu et al. (18) reported a novel “hands-as-feet” teaching method that can more effectively help medical students and young medical workers understand complex anatomical relationships and variations, making the teaching process more visualized.

## Conclusion

We report a case of a 23-year-old lady who underwent endoscopic thyroid surgery via total areola approach for radical resection of bilateral thyroid cancer and a NRLN was encountered intraoperatively. Although the incidence of NRLN is low, the risk of iatrogenic injury is high if this anatomical anomaly is not taken seriously. This case highlights this anatomical variant. If no RLN is found after meticulous dissection in the usual location, combined with preoperative radiologic analysis (CT, ultrasound), NRLN should be highly suspected.

## Data Availability

The original contributions presented in the study are included in the article/Supplementary Material, further inquiries can be directed to the corresponding author.
